# The Apgar score and race: why healthy babies are supposed to be “pink”

**DOI:** 10.1007/s40656-025-00693-3

**Published:** 2025-10-09

**Authors:** Rebecca L. Jackson

**Affiliations:** Department of Philosophy, Institute of Medical Humanities, Durham University, Confluence Building, Stockton Rd, Durham, Durham DH1 3LE UK

**Keywords:** Philosophy of measurement, History of measurement, Patient outcome measures, Apgar Score, Coordination, Validity

## Abstract

Outlining the Apgar Score’s use throughout the latter half of the twentieth century, I propose that the historical abuse of this score for newborn wellness does not only come from the obviously white-centered assessment criteria for “color” established in the 1950s. The more concerning aspect of the Score is its potential interpretation as measuring one unitary construct which captures both the past asphyxiated condition and future health risks of individual infants (a problem that has been noted for decades in professional guidance documents). My novel contribution is to use the history of the Apgar Score’s use and misuse to demonstrate why racial inequities in medicine pose a problem for two frameworks in philosophy of measurement when applied to patient outcome measures. I ultimately argue that the case of the Apgar Score shows how both dominant frameworks in philosophy of measurement, that of coordination (within the representational theory of measurement) and that of psychometric validity, fail to help us fully comprehend the challenge of clinical measuring with indices. Both frameworks expect that, at some point, the process of coordination or validation of an instrument will end. An expanded and historically-informed framework is warranted for understanding how patient outcome measures are validated (and re-validated) over time, which can include the social and institutional forces which render an index relevant, biased, or questionable for different aims.

## Introduction: measuring “pink” babies

The event of birth is marked by many kinds of quantification, from the number of hours in labor to measuring the height and weight of the infant. These quantities have become part of cultural meaning we assign to childbirth in addition to epidemiological data for later analysis. Readers born after the 1970s may very likely have received another kind of quantification at their birth: a “Score” from 0 to 10. Most are never made aware of the existence of this datapoint, let alone how well they scored. Yet, the “Apgar Score” influences the actions of medical professionals just minutes after an infant emerges into the world, and this number is based at least partly on an assessment of the infant's skin tone (the “Appearance” sub-score). In this paper, I explain the following: (1) Why the “Appearance” item of the Apgar Score is not as worrying as it initially appears (although the wording for healthy skin tones, “pink,” is white-centric, inaccurate, and far overdue for change), (2) why the more concerning aspect of the Score is its potential interpretation as measuring one unitary construct which captures both the past asphyxiated condition and future health risks of individual infants (a problem that has been noted for decades in guidance documents), and (3) why racial inequities in medicine pose a problem for two frameworks in philosophy of measurement when applied to patient outcome measures. This paper represents a new way to conduct “critical metrology” in medicine,^1^ expanding on critical medical humanities approaches to measurement studies (e.g., Malpass et al., 2022; Swyer, 2024). Previous approaches to critical metrology tend to focus on uncovering the ways that (supposedly objective) measures negatively impact marginalized groups or prioritize the needs of privileged groups. Rather than aiming to critique *the validity of the Apgar Score* as a fair measure, ultimately, my argument pertains to theoretical framings of *validity* itself. I argue that the assumptions inherent to both dominant philosophical frameworks for arriving at sound measures (psychometric validity and representational coordination) are troubled by the racialized realities of the clinical context and cannot explain the historical trajectory of the Apgar Score.

The Apgar Score is a formative index for assessing the overall condition of newborn babies 1-min and 5-min after birth.^2^ This index arose from a method of scoring developed by Virginia Apgar from 1949 to 1952 for use in obstetric anesthesiology research (Apgar, 1953), and later became repurposed as a clinical guide (as well as serving epidemiological purposes). The scale of this Score consists of ordered whole numbers from 0 to 10, and is formed by summing each of five sub-scores (0–2) (see Table 1).

**Table 1:** APGAR scoring table, adjusted to reflect inclusive and accurate terminology for “appearance”

SIGN	0 Points	1 Point	2 Points
Appearance	Blue/pale all over	Blue extremities, flesh-toned body	Flesh-toned all over
Pulse	Absent	< 100 bpm	> 100 bpm
Grimace	No response	Grimace	Cry on stimulation
Activity	None	Some flexion of arms and legs	Active flexion
Respiration	Absent	Weak, irregular, slow	Strong, crying

Each sub-score is intended to capture a readily apparent aspect of the infant’s overall physiological condition: Appearance (skin tone), Pulse, Grimace (response to stimulation), Activity (muscle flexion), and Respiration. These five “signs” are designed to enable an immediate assessment of whether an infant is at risk of extended asphyxiation, which could risk neurological damage. A sub-score of “0,” “1,” or “2” is assigned for each sign, with “0” being the worst condition. For example, a “0” in Appearance would be noted if the baby’s entire body appears to be a blueish tint (called “cyanosis,” a signal the blood lacks adequate oxygen). The best Appearance score, “2,” is assigned when the entire body is a reddish flesh-tone all over. If the core of the body appears to be flesh-toned, but the extremities (limbs) are blue, a middling score is earned (“1”). The overall Score is totaled at 1 and 5 min after birth, with lower scores indicating that the infant requires extra vigilance from care providers. In more extreme cases, infants who continue to score low may require oxygen therapeutic interventions and even intubation. The current guidelines support interpreting a 5-min Apgar Score of 7–10 as reassuring, a score of 4–6 as moderately abnormal, and a score of 0–3 as low for most full-term infants.^3^ Infants which fail to achieve a “reassuring” score after five minutes will continue to have their score recorded every five minutes for the first 20 min (even during active resuscitation).

In the table above (Table 1), I chose to make a slight adaptation of the Apgar Score as it is usually presented. In the vast majority of teaching materials used in the US today, instead of “flesh-toned” (as I have listed above), the word “pink” is used. While infants vary in the level of melanin their skin starts out with (melanin production increases over the course of the first three weeks, to protect the skin), it is fair to say that this descriptor seems to expect that babies will be on the low end of the melanin spectrum. Most doctors do not consider the term “pink” to be a good descriptor of healthy African American newborns, for example (Adams & Grunebaum, 2014). The centering of babies with pale skin is, clearly, unjust. This is a particularly urgent issue in the US context, where we know that African American mothers and infants see much worse risks of morbidity and mortality compared to White women. In 2020, for example, the infant mortality rate for non-Hispanic Black infants in the US was twice that of non-Hispanic White infants (NCHS, 2022). Maternal mortality disparity in the US is even worse; non-Hispanic Black mothers saw a rate nearly three times as high as non-Hispanic White mothers in 2020 (Hoyert, 2022). While many social and economic factors contribute to disparities in the availability of quality care, medical training which centers whiteness (including the terminology and exemplars for what signs to look for in “normal” or pathological conditions) also proliferates these inequities (deShazo et al., 2021; Plaisime et al., 2023). Due to the urgency of the current maternal–fetal mortality crisis and the existence of empirical evidence of racialized health disparities, my arguments in this paper focus mostly on the historical use of the Apgar Score in the United States. Although the Score is used globally, it originated in the 1950s US-American cultural context and this origin still has global impacts today.

Outlining the Apgar Score’s use (and abuse) throughout the latter half of the twentieth century, I propose that the abuse of a score for newborn wellness does not only come from the obviously white-centered assessment criteria for “Color” (now called “Appearance”) established in the 1950s, but also from broader misapplications: First, the misuse of a research tool (Apgar’s “scoring method”) as a clinical one, and later the misuse of a clinical tool (the “Apgar Score”) as a statistical predictor of an individual infant’s future intelligence and behavior. Once the content of the Score is adjusted to better describe the appearance of all healthy infants and ceases to be misinterpreted as a crystal ball foretelling individual infant futures, our real work begins: How can we be sure that the Apgar Score is operating equally well for all infant racial groups? One recent study found a “Reduced strength of association between the 5-min Apgar score and mortality in black and non-Hispanic non-Asian groups,” meaning the Apgar Score was a better predictor of mortality for white non-Hispanic infants (Gillette et al., 2022, p. 11). The disparity is there, but whether the Apgar Score is capturing an already-present disparity, or adding to the disparity, is more difficult to ascertain.

This line of inquiry highlights the inadequacy of validating a clinical measure within the framework of psychometric theory,^4^ which traditionally takes for granted that the research which validates the measure can be accomplished outside of the environment in which it will be implemented. This is not an assumption we can generally take with clinical measures. I ultimately argue that the case of the Apgar Score shows how both dominant frameworks in philosophy of measurement, that of coordination (within the Representational Theory of Measurement) and that of psychometric validation, fail to help us fully comprehend the problems of clinical measuring with indices. Psychometric validation, briefly defined, is the process of assessing whether a score measures what it purports to measure.^5^ Both frameworks expect that, at some point, the process of coordination or validation of an instrument will end. An expanded framework is warranted for understanding how patient outcome measures are validated (and re-validated) over time, which can include the social and institutional forces which render an index relevant, biased, or questionable for different aims.

In the following three sections, I outline the various shifts in how the Apgar Score has been used during the twentieth century (see Table 2 in Appendix for an overview of the main phases). First, I discuss its beginnings in the 1950s as Virginia Apgar’s scoring method, a research tool for assessing anesthetic techniques (Sect. 2). Then, I outline its transformation into the “Apgar Score,” which consisted of two scores (at 1-min and 5-min) utilized in clinical and research applications in the 1960s (Sect. 3). During this period, it played a dual role. It became an operationalization of clinical observations (and a rule of thumb) for prompting attention and guiding clinical action and triage (regarding individual babies), meanwhile acting as a mediator of risk assessments (of groups of babies) for research purposes. Due impact of the Collaborative Perinatal Study (1959–65), Apgar Scores became more widely used and came to be seen as recording the status of individual babies and their risk of future mental defects. In the following decades, this enabled two misuses: Scores used as diagnostic (of asphyxiation) and retrospectively prognostic (of neurological damage due to asphyxiation). Throughout, I explain the ways that race did (or did not) participate in arguments justifying the Score’s validity for these various uses, and why the misuses of the score (diagnostic and prognostic), while seeming to apply to all babies, may have had racialized impacts. Section 4 outlines how this history exposes the shortcomings of two broad ways of thinking about how meaningful measurement is achieved: representational coordination and psychometric validation.

## Origins of Apgar’s scoring method: enabling babies to measure their doctors

Virginia Apgar’s scoring method was originally designed (between 1949 and 1952) to enable anesthesiologic research with a clinical motivation in mind: to make doctors pay attention to newborns. Apgar, trained as a surgeon, turned to anesthesiology and obstetrics as an area where women physicians could make a significant impact on a nascent area of medical research (albeit, not much money). Anesthesia had become a regular part of labor and delivery, with nurse anesthetics (and later, anesthesiologists) playing an active role in sedating and resuscitating the mother when necessary. The problem Apgar saw was that none of the medical specialists present at a birth were trained to do anything with *the infant*. After the umbilical cord was cut, the obstetrician was focused on the rest of the labor (delivering the placenta), the anesthesiologist (when available) was primarily there for the mother, and the pediatrician was often elsewhere, waiting for the child to be brought to them. Apgar suspected that this was why the rate of infant deaths in the first 24 h remained stubbornly high, despite steady improvements in overall mortality rate in recent decades (Apgar, 1950). She believed that if struggling newborns were noticed, and if resuscitation techniques were appropriately employed to revive oxygen-deprived babies, some of those lives could be saved.

From biochemical studies, it was known that the level of oxygen in newborns’ blood could vary greatly. Yet, there was no evidence that showed conclusively whether asphyxiation at birth was a pathological state, rather than just a product of random and natural blood-gas variations among neonates (newborns) (Apgar & James, 1962, p. 139). From her clinical experience training under an elite research group of anesthetists (the “Aqualumni” of Ralph Waters) (Aqualumni, 1942), and under nurse anesthetists at Columbia Presbyterian’s Sloane Hospital for Women, Apgar suspected that some of the anesthetic drugs given to mothers were making their way across the placental barrier and depressing the infant’s physiology. Her eventual proof of this would not come for several more years (Apgar et al., 1957); in the meantime, babies were still being born. Guiding anesthetic practices by infant outcomes was one goal she had early in her career. Identification of infants in need of respiratory intervention was another. But, her first (and primary) battle was for attention: She needed to prove that newborns needed to be observed immediately, as respiratory therapy must be done on the scale of minutes or (preferably) seconds to be effective. In order to do this, she needed to first prove that babies were not supposed to be born asphyxiated. The fact that it now seems absurd to argue otherwise is, in no small part, thanks to Apgar.^6^ Her scoring method, which represented clinical observations of infants’ physiological depression with a number, was a way to make in-roads on both fronts. She used it to show that levels of depression could in fact depend on anesthetic techniques. At the same time, this allowed her to argue (based on the score differences) against techniques that unnecessarily impacted newborn physiology.

Unlike the version of the Apgar Score we know today, presented in a convenient table with sparse wording, Apgar’s first published paper on her scoring method describes each sign in detailed paragraphs. This reflects Apgar’s motivation in creating the score to begin with: it was a way to conduct research on the impacts of techniques and interventions of anesthesiologists, using structured clinical observation of newborns to judge best practices. Much like one might present a “Methods” section in a research paper, she includes details about how scoring conventions were established and what limitations she identified with her method. Of her five signs (at the time, termed “Heart Rate,” “Respiratory Effort,” Reflex Irritability,” “Muscle Tone,” and “Color”) the least satisfactory in her view was the latter, “Color.” This sign caused “the most discussion among observers” when they were trying to assign a score (Apgar, 1953, p. 261). First, a newborn’s skin is obscured by vernix—a white, waxy substance which coats the fetus in the womb. Additionally, Apgar noted a racialized difficulty with assessing blueness (cyanosis) due to the “inherited pigmentation of the skin of colored children” (more on this below). Lastly, it was unusual for any child to be scored as a “2” (given only when the “entire child was pink”), as almost all children were at least somewhat cyanotic for the first minute of life. Although they recorded the score at one minute, as this had been determined to be the most “practicable and useful” timing for clinical purposes (Apgar, 1953, p. 260), Apgar noted that the Color sign seemed to improve almost universally after five minutes.

One might wonder why, after noting so many problems with the “Color” sub-score, Apgar did not simply drop it from the list or replace it with another sign which produced less disagreement among doctors. My best explanation for this is two-fold: First, the Color sign had clinical advantages that outweighed its ambiguity. According to Apgar, it operated as a delayed snapshot of the primary signs in her list (Respiratory Effort and Heart Rate) (Apgar, 1953, pp. 261–262). A low Color sub-score signaled that an infant had been without adequate oxygen for some time—that is, that the Respiratory Effort and Heart Rate sub-scores had also been low moments before. This allowed for a practitioner to distinguish between a baby who had only just started to struggle from one which had already been struggling. Likewise, a low Color sub-score along with high Respiratory Effort and Heartrate scores showed that the infant’s condition was improving. This temporal relationship between the sub-scores may have been an inspiration for the five-minute score she later adopted in addition to the original one-minute score. All in all, it seems that the clinical utility of the Color sign took precedence over abstract notions like inter-scorer reliability (doctors assigning the same score for the same observation).

**Fig. 1:**
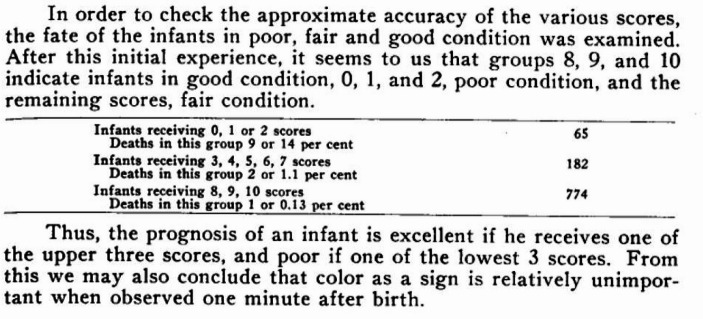
Infant Deaths by Score Groupings of 0–2, 3–7, and 8–10 (Apgar, 1953, p. 267)

Second, the flaws of the “Color” sign did not significantly affect her analysis. She thought of her score as a more structured and meaningful way to code clinical observations of the baby’s physiological state than simply assigning broad categories of “mild, moderate, and severe” depression as had previously been done (Apgar, 1953, p. 260; Eckenhoff et al., 1952). The actual data she was interested in is better thought of as these broader status differences, with scores serving as a method for arriving at these categories. This is evidenced by how the analysis she did to “check the approximate accuracy of the various scores,” which she did by making aggregated comparisons of the percentage of deaths sign for the most extreme score categories, 0–2 and 8–10 (see Fig. 1) (Apgar, 1953, p. 267).

She notes that, in light of the huge mortality differences observed, the flaws of “Color” did not matter much. This is also apparent in her analysis of anesthesia methods, which considers only large average score differences to be indicative of a difference between anesthesia methods (p. 265). Whether a particular baby was a “7” or an “8” did not matter so much as comparing the overall picture of how babies tended to fare after an intervention. For example, infants whose mothers delivered by c-section under spinal anesthesia scored an average of 8.0, whereas those whose mothers received general anesthesia instead scored an average of only 5.0 (Jackson, 2023, p. 185; Apgar, 1953, p. 263).^7^

In summary, the score was designed to enable comparisons of interventions, not to make an exact measurement of babies. It allowed anesthesiologists to use babies as their own measuring stick, to identify which of their techniques fell short and in what cases. The Score was more transparent and well-defined than previous so-called objective methods of measuring infant outcomes, such as using “breathing time” or “crying time,” or vague degrees of “depression” (Apgar, 1953, p. 260; Hapke & Barnes, 1949). And, most importantly, it directed attention to the importance of examining newborn infants, and codified neonatal observation as an area of medicine that one can and should have expertise. Apgar succeeded in making an area of anesthetic and resuscitation practices testable—taking the tacit knowledge that nurse anesthetics employed and transforming it into a basis for conducting Anesthesiology research.

Apgar’s reasons for not being concerned about the ambiguity of the “Color” sub-score for anesthesiology research in 1953 still apply today when considering the clinical use of the score on individual babies and the epidemiological use of the score (on groups of babies). When employed properly and according to current clinical guidelines, and if inclusive language (such as “flesh-toned” or “flushed”) is used to describe healthy babies, we have little to fear from the ambiguity of the “Appearance” item in the score. First, as Apgar noted, every baby’s skin is obscured by a white waxy substance—this is the main obstacle to quickly assessing cyanosis. Additionally, the vast majority of babies (regardless of race) start out much paler than they will eventually be in maturity; melanin production begins to increase in the first few weeks to protect the skin from the sun (Carter et al., 2023, p. 526). For babies who start out life with higher levels of melanin already present in their skin,^8^ according to current guidelines, a single point difference should not make or break any clinical decision-making. The one-minute Apgar Score, properly considered, is a heuristic for guiding clinical observation of individual babies; it should serve only as a rule-of-thumb for drawing attention to infants who are very clearly in need of aid.

As will be outlined in the next section, Apgar’s scoring method did not remain a way for babies to measure their doctors’ techniques, as it was in 1952. In the course of the next two decades, it became the “Apgar Score,” which consisted of two scores (at 1-min and 5-min) utilized in clinical and research applications. The uses of the Score multiplied as it was exported outside of anesthesiology and outside of the birthing room. In the decades after Apgar’s death, Scores assigned to individual babies were recorded, preserved, and subsequently used as evidence of them having experienced a physiological condition (and even as evidence of downstream effects of prolonged asphyxiation). This logical error, an instance of the ecological fallacy (Robinson, 1950/2009), results from conflating the significance and predictive value of data at the group-level as having the same predictive value for individuals. Another term for this is the “G2i” or group-to-individual inference problem, used in the legal context to describe the difficulty of interpretating how and whether statistical data may apply to a single case (Faigman et al., 2014). This is not to say that all uses of the Apgar Score are problematic—see Table 2 in Appendix for clarity on which uses (in bolded text) I see as committing the ecological fallacy and which uses seem in line with how the Apgar Score can and should be used.

The following section will recount how professional guidance documents for the Apgar Score in obstetrics have noted this issue (of conflating statistical evidence about groups of infants with diagnostic evidence for a past condition of a single newborn) for decades. The Apgar Score cannot be used to diagnose infants with asphyxiation or other maladies retrospectively at the individual level; regardless of the race of the individual, this is a grave misuse. Yet, racial biases may make certain babies more likely to be subjected to this logical error, and the impacts compound with other difficulties experienced by marginalized communities. This is the true source of injustice in the historical abuse of the Apgar Score.

## Mistranslational medicine: abuses of the Apgar Score from 1960s–today

The historical injustice of the Apgar Score goes far beyond the obviously white-centered assessment criteria for “Color” established in the 1950s. The more serious (and subtle) harm has come from a broader misapplication of a research tool (Apgar’s scoring method) as a clinical tool for retrospective diagnosis, and later a clinical tool for triage and rapid treatment (the “Apgar Score”) as a statistical predictor of an individual infant’s future intelligence and behavior. As mentioned in the previous section, Apgar originally designed her scoring method as a way to assess the impacts of anesthetic and obstetric techniques and interventions on newborn wellness. In effect, Apgar’s method enabled groups of babies to act as the measuring stick by which anesthesiologists could identify best practices. This was a part of her longer-term goal of showing that levels of asphyxiation were not randomly distributed among newborns, and that respiratory interventions could actually save struggling newborns from unnecessary harm. Rather than directly assess infants as having “mild,” “moderate,” or “severe” degrees of depression, scoring was a method for systematically assigning infants to groups of outcomes that could be easily interpreted as clearly preferable or clearly not preferable. To evidence the fact that these infant groups did in fact differ in their health status, she compared number of deaths in the most extreme score categories (0–2 and 8–10) to show that many more deaths occurred among babies scored as “poor” than babies who scored as “good.”

In the late 1950s, with the help of L. Stanley James and others, Apgar finally achieved her dream of using the Score to show that infants were not supposed to be born asphyxiated. The Apgar Score aided the interpretation of biochemical data, by showing that blood-gas levels were associated with Apgar Score (Jackson, 2023, pp. 214–221). If a low Apgar Score could be impacted by alterations in obstetric and anesthetic practices, then so could the infant’s asphyxiated condition. At the same time that the Apgar Score was being used for neonatal physiological research, it was also being repurposed as a clinical tool. Apgar used evidence from biochemical studies to reverse-engineer the Score for use in guiding clinical action and triage. No longer was the Score a mere reflection and refinement of clinical judgment, making the observations of nurse anesthesiologists legible and aggregable for research purposes. The Score could be used as a rule-of-thumb for the inexperienced, informing them when infants needed closer observation or, for very low scores, when the dangers of intubation might be worth the risk compared to the neurological damage that could result from lack of oxygen to the brain.

Over the course of the 1960s, the Apgar Score became a mediator of risk assessment. Instead of infant mortality being used as a “gut-check” for interpreting loose score-groupings as roughly representing babies in “poor” vs. “good” categories, the Score began to represent of a level of mortality risk itself. If three times as many deaths were found to occur in “5”-scored babies as compared to “8”-scored babies in general, this was used as a mediating variable for interpreting the risk of death inherent in a course of clinical action. For example, if an anesthetic technique resulted in babies scoring an average of “5,” this technique was read as having a higher mortality risk than an alternative which led to babies to babies being scored as “8” on average (Jackson, 2023, pp. 197–210). During this period, there was a gradual transformation in how the Apgar Score was used and interpreted—as *measuring babies*, rather than Scores enabling babies to become a measure for other things. This reversal was enabled by the monolithic, nation-wide Collaborative Perinatal Study across 12 top-tier American birthing institutions, which tracked the development of over 17,000 babies (in the initial cohort, 1959–1965). This study included the Apgar Score among many other variables related to birth and early childhood development. Reports on the resulting data confirmed Apgar and James’ previous claim that there was an association between Apgar Score and infant mortality (Apgar & James, 1962; Drage et al., 1964), as well as established the Apgar Score as an epidemiological predictor of neurological damage due to asphyxiation (Drage et al., 1966).

As the distance between the Apgar Score and Apgar herself began to grow, she felt the need to push back against some of the misuses she foresaw occurring from broadening scope of the Score’s application. The Collaborative Perinatal Study also showed that the 1-min score and 5-min scores seemed to diverge in their association with eventual infant outcomes. The 5-min score was a better predictor of long-term damage and death—suggesting that the 1-min score may be less important. Apgar preemptively struck back: to drop the 1-min score was to all but guarantee that more data points of death and disability would result (Apgar, 1966). The 1-min score, Apgar argued, was naturally going to be less associated with bad outcomes. After all, the hope was that rapid scoring would direct clinical attention to babies at risk of poor outcomes would lead to actions averting these outcomes altogether. The poor association of the 1-min score with death showed the Score’s clinical utility, that is, it showed exactly how important it was to pay attention and intervene early. The best clinical measures serve a heuristic role as well as an epistemic role, directing practitioner attention to the right information at the right time (Jackson, 2021).

History shows that only half of Apgar’s advice was adhered to. Both the 1-min and 5-min Scores were indeed preserved as routine practice. Unfortunately, after the Collaborative Study had shown associations with mortality and neurological impacts from asphyxiation, the Score became reinterpreted causally. No longer was the Score a mere operationalization of clinical observations and a rule of thumb. Its role in research, previously, had been to reinforce the importance of its clinical role in directing attention and action. Instead, the Score became used as an explanation for negative events in an individual child’s development. In every decade since 1986, American College of Gynecology and Obstetrics (ACOG) and American Academy of Pediatrics (AAP) have published revised recommendations for reigning in the interpretation and use of the Apgar Score in clinical research and practice. In its 1986 committee report on “Use and Abuse of the Apgar Score,” the AAP sought to correct the tendency to interpret the Score as a diagnosis or definition of asphyxia itself, rather than as “a convenient shorthand for reporting the state of the baby” (AAP, 1986, p. 1148). The Score was also being used for “prognostication of future neurologic deficits,” a use Virginia Apgar had discouraged (ibid.).

The mistaken use of the Apgar Score for predicting “future neurologic deficits” was not limited to early childhood development; scores could follow children far into adulthood. For example, still using data from the Collaborative Perinatal Project 30 years later, one behavioral psychology study primarily targeting Black youth in Philadelphia cited the interaction (co-occurrence) of mothers smoking and low Apgar Scores as a significant cause of eventual criminal behavior (Gibson & Tibbetts, 1998). The Apgar Score, misused as a diagnosis of asphyxiation and neurological damage, played a role in explaining how smoking mothers set their children on the wrong foot. Their unhealthy infants were born (supposedly) deficient in their capacity for moral fortitude. The temptation to explain crime rates in terms of measurable mental deficiencies in individual people, rather than being rooted in poverty and inequality, has existed in the US since at least the late nineteenth century. With the rise of the eugenics movement in the twentieth century came the scientific examination of “feeble-mindedness,”^9^ as catch-all term that gave a common explanation to intellectual disability, biological inferiority or defect, and moral weakness (Katz & Abel, 1984). In the 1990s, rising crime rates in the US (racialized as a problem belonging to urban areas) gave new impetus to uncovering why crimes are committed. The most valuable answers, for those who hoped for solutions that did not require an increase in welfare spending, redirected attention to the actions of individual people—often, mothers. The “bad” mothers who smoked during pregnancy were blamed for the neurological deficiencies of their supposedly morally weak children.^10^ The Apgar Score and maternal smoking, together as an interaction variable, became interpretable as a seemingly scientific explanation for inner city Black youth delinquency.

Future historical and sociological work should be done to uncover the stories of how these impacts have been experienced by individual youths and parents. If racial bias in healthcare, educational, and legal systems created a similar compulsion to find intrinsic (rather than systemic) reasons for *individual* youths’ behaviors, the Apgar Score may have served as an excuse to dismiss external factors. For example, a child’s struggle to learn could be conveniently interpreted as an inevitable result of a low Apgar Score, rather than any number of other factors (disengaged instruction, being a target of bullying, boredom, or the need for more physical movement).^11^ It is an open question whether (and how) the Apgar Score has had a racialized impact on those navigating systems which are demonstrably biased against them. It is important to note that, if this kind of biased interpretation of Apgar Score has widely taken place, this would be a racialized use that could have little to do with skin tone at birth, as racialization can take place regardless of skin tone.^12^ Those of us who have never known our own Apgar Score should count ourselves lucky that our neurodivergences and behaviors have not been attributed to a number which was decided minutes after birth and cannot be amended.

In 1974, the same year that Apgar died of pancreatic cancer, she marveled that the Score was being tested for its associations with so many variables seemingly unrelated to its origins in directing attention to newborns: from IQ at school age, to behavioral disorders, Tay-Sachs (a fatal infant disease), and even autism (Apgar, 1974). Although Apgar and her colleagues had, in the early 1960s, tested associations of lower Scores with negatively impacted IQ, this was simply a method for proving that asphyxiation was in fact not a natural state of newborns and that non-intervention impacted children’s safety. Reading the association of asphyxiation and Score backwards, and interpreting the Score as a causal predictor of intelligence or behavior for individual infants, did not appear to be a fruitful line of inquiry in Apgar’s view. It was not until 2006 that the ACOG and AAP arrived at a similar stance: the Score had been “used inappropriately to predict specific neurologic outcome in the term infant” (ACOG & AAP, 2006). This shift was at least partially due to better understandings of asphyxiation itself,^13^ which further confirmed Apgar’s interpretation of the Score. Rather than a measure of asphyxiation, the Score was better thought of as an outcome measure that shows what generally happens to babies when asphyxiation is allowed to continue. In the ACOG/AAP’s 2015 guidance, the language warning against Score misuse became even clearer about the dangers of misapplying the kind of information we can know about large groups to prognosticate about the future development of individual babies: “it has been inappropriately used to predict *individual* adverse neurologic outcome” (emphasis mine) (ACOG & AAP, 2015).

While it was Virginia Apgar’s hope that “no harm” would come from research that tested “under what conditions the score is useful or useless” (Apgar, 1974), it appears that this was a bit too optimistic. The fact that the Apgar Score is used in legal argumentation means that it is still being employed to represent biological states of individual infants (Brown Trial Firm, 2023; McCready Law, 2021, Cleveland Birth Injury Lawyers, 2023). To a medical care provider in the moments after birth, a score difference of a point (or even two) should not make much difference in their decision-making. Regarding individual infants, the Score’s only proper use is in offering a systematic heuristic for directing clinical attention and action. However, if this score is recorded and later interpreted by a lawyer, an insurance company, a school guidance counselor, or a psychologist, they may interpret the score as a recorded fact about an individual infant’s biological state. Knowing the history of the Apgar Score should lead us to reinforce its proper place: It should never be used to say something retrospectively about individual babies, regardless of their race. In the clinical context, where the Apgar Score has a proper role for guiding observation of individual infants (as discussed in Sect. 2), we should expect that medical practitioners be well-educated in identifying the full range of flesh-tones in newborns. For the Apgar Score to serve as an ethical and epistemically meaningful guide for clinical decision-making, even as a rule-of-thumb, we should demand that obstetric professionals be given inclusive language (like “flushed” rather than “pink”) and first-hand experience with babies from different racial groups that exhibit the different Appearance sub-scores in their training. In the research context, as in judging the best care practices or evaluating national health systems wherein the Score is properly used to measure *large groups of infants*, issues with the Appearance subscore, in isolation, should have much less impact. Small differences in Score assignment should have little effect on broad evaluations of what practices help or hurt infant children more broadly. Yet, in reality medical practices are never in isolation from broader social behaviors and biases. Section 4 will thus address how and why it is difficult to confirm whether misinterpretations of the Score as retrospectively representing the state of an individual infant could in fact still trouble epidemiological research. We do not currently have an adequate philosophical framework which allows us to tackle this question in more than an idealized or aspirational form, which does not help us within the racially unjust health system in which our answers to this question must apply.

## The epistemic problem within the ethical failure: how racialized health disparities impact outcome measure assessment

I have argued that what appears to be the problem with the Apgar Score (the white-centered language for the “Appearance” sign) is actually low-hanging fruit. We can hope to soon live in a future where medical care providers employ more inclusive language to describe healthy babies; I demonstrated a possible adaptation of the usual language for the appearance subscore in Table 1. The deeper problem with the Apgar Score is its misinterpretation as a cause (or as an explanation) of individual children’s health outcomes, behaviors, and neurological capabilities. Yet, as noted in the previous section, this is a problem which is becoming increasingly acknowledged by pediatric and obstetric professional bodies, since at least 2015. If the most recent guidance shapes medical education (AAP & ACOG, 2015), we will see fewer mistranslations of the Apgar Score into retrospective diagnoses which cannot be made based on Scores. Yet, there is one further question we can ask about the use of the 5-min Apgar Score for epidemiological purposes. Putting aside small changes to terminology, does the Apgar Score operate equally well when used statistically across racial groups? If the same systematic biases that have been noted for neonatal pulse oximetry (wherein oxygen saturation is systematically over-estimated for babies with more melanin (Sharma et al., 2024)) also exist for the Apgar Score, this could affect interpretability of statistical data and comparability of scored populations.

As mentioned in the Introduction, we already know that Apgar Score distributions differ across racial groups in the US. Yet, because there are real differences in health outcomes between these groups (including difference in rates of infant death due to asphyxiation, see Javeid et al., 2025), this difference unto itself is not necessarily evidence that the Apgar Score itself is racially biased. In fact, we would expect to see a disparity. This problem is not unique to the Apgar Score, and impacts patient outcome measure assessment more generally. While philosophers have begun to pay more attention to the epistemic problems faced when developing outcome measures in medicine (McClimans, 2010), known racial health disparities compound these issues within two broad ways of thinking about how meaningful measurement is achieved: coordination and validation. First, I discuss challenges from the perspective of coordination, and then from the perspective of psychometric validation.^14^

### Problems when coordinating patient outcome measures

Measurement as a process of coordination is usually associated with the Representational Theory of Measurement (RTM).^15^ The RTM imagines measurement as the process of establishing many-to-one mappings from a set of empirical observations of a property with a set of representative notation that reflects the relationships between these observables. For example, representing weight in numerical units with additive structure (1 kg, 2 oz, etc.), to reflect the fact that weight itself is an additive property (i.e., two weights which measure 1 kg each will, together, weigh the same as a 2 kg weight). History shows that this coordination is rarely a straightforward task, even with relatively “simple” physical properties; the classic example of coordinating temperature with ratio-scale numerals took over 200 years. We never start with a perfect instrument or a perfect theoretical understanding of what it is exactly that we are measuring. In order to get closer to having either, we start with rudimentary versions of each which must mutually correct one another over long periods of time (epistemic iteration) (Chang, 2004). The framework for coordination in RTM, which was originally derived from ahistorical examples of measurement in physical sciences, is limited in its capacity to describe the material, iterative way coordination is actually achieved (Frigerio et al., 2010, pp. 130–131). Charitably interpreted, RTM is “an abstract theory of the kinds of well-behaved scales that one encounters [already constructed] in science” (Luce & Narens, 1994, p. 223 [insertion mine]). Thus, today this view is best understood as an idealized model or goal, or perhaps a helpful heuristic (Tal, 2021), rather than an actual description of how measurement is achieved in science.^16^ It has also been framed as an epistemic problem scientists attempt to solve, the “problem of coordination” (Barwich & Chang, 2015). But, even taken as merely a description of where we hope to eventually land with a successful measuring practice, it has limited theoretical language for describing how we know we have arrived at coordination,^17^ let alone the particular perils (and advantages) of outcome measure assessment in the clinical context.

There are some features of the Apgar Score’s history that are similar to sophisticated accounts of achieving representational coordination, like that of temperature. After all, the Score was first designed to loosely represent categories of infant wellness in order to prove a theory (that asphyxiation was not a natural state and should be ameliorated). In turn, that advance in theory refined the use of the Apgar Score, allowing for rules-of-thumb and cut-points within the scale to aid decision-making. Similarly, understandings of asphyxiation improved; the term “asphyxiation” conflated both causes and effects of oxygen deprivation, and finer distinctions were thus warranted and the relationship to the Apgar Score refigured accordingly (Jackson, 2023, p. 237). And so, we would expect that, over time, the Score would become more and more coordinated with one well-defined biological or physiological dimension, such as umbilical cord blood pH level, or perhaps metabolic acidemia. Instead, clinical usefulness trumped chemistry: blood gas levels turned out to be worse predictors of infant morbidity and mortality than Score, at the aggregated level. At the individual level, the Score was faster than a blood test. In the time-sensitive context of birth, a slow measure would not be a measure of the right thing. Unlike in the story of temperature,^18^ the Score bifurcated into two scores: The 1-min score for rapid clinical use, and the 5-min score for subsequent aggregated research uses. What instructed this multiplication of scores, coordinated with clinical uses rather than coordinated with well-defined biochemical dimensions? Why did the Score not become better and better coordinated with some biochemical datum, or simply become obsolete when this coordination failed? As we know from Sects. 2 and 3, the Score instead became coordinated with sets of infant outcomes and clinical actions that can improve these outcomes. To understand the process of coordination that the Apgar Score underwent requires understanding that, in addition to clinical outcome measures facing distinct challenges, they also have epistemic advantages.

One advantage of measurement in the clinical context is that there are strong auxiliary theories (see Trafimow, 2012) that can guide the measuring process. Signs from the human body—in the most extreme case, patient death—provides physiological information outside of the measuring process that can tell us something about how to achieve better coordination.^19^ Not always are we so lucky in measurement, although the role of auxiliary theories in coordination is not unique to the clinical context.^20^ Underlying the trust in any patient outcome measure are higher-order patient outcome measures,^21^ often taking advantage of these strong auxiliary theories—but they are always *worse* for the purpose at hand. Otherwise, there would be no reason to develop the lower-order patient outcome measure. Sometimes what makes the higher-order outcome measure “worse” is that it costs us too much, financially or ethically. For example, if the goal is to avoid patient death, the hope is that over time that source of evidence becomes vanishingly rare. Sometimes the higher-order outcome measure takes too much time, and data cannot be produced fast enough to keep pace with the questions that are being asked. In the case of the Apgar Score, arterial blood gas analysis was too expensive and time consuming to answer the questions Virginia Apgar wanted to ask at the time they needed to be answered. Importantly, what makes a higher-order patient outcome measure trustworthy for assessing the lower-order outcome measure is that it has other valuable epistemic features, such as better face validity.^22^ Sometimes this comes from having a scale which is more obvious to assess from physiological evidence, such as a binary categorical variable of “dead” or “not dead.” While this is not always an easy call to make (Goodwin, 2018), most of the time we do not feel the need to report quantitative evidence from an even higher-order outcome measure (such as pulse in BPM) in making this determination.

The epistemic advantages that we have when coordinating clinical measures like the Apgar Score are muddied by social inequities. The problem with using higher-order evidence (like number of deaths) to achieve coordination in a systemically unequal society to assess whether the measure works equally well for different racial populations is that the higher-order outcome measures, if working correctly, can be expected to demonstrate these health inequities. In fact, if the Apgar Score showed identical distributions for racial subpopulations that we know have divergent health outcomes more generally, this evidence could suggest that the Score might actually in fact be biased. This is a case when, to use psychometric language, we would actually *not* take intergroup reliability (similar assignments made across groups) to translate to supporting overall validity of the measure across racial groups.

From the perspective of coordination, we could say that what truly matters is that the level of association (e.g., how well the Score correlates with mortality figures) across racial groups remains the same, rather than having the same distribution of scores across racial groups. But, we would need to assume that an observed divergence in the level of association between Scores and mortality is due to the divergent performance of the Score, and not other factors (beyond physiology) that could lead to infant death (such as differential medical treatment (Hall et al., 2015)).

For example, in 1964, a report from the US-wide Collaborative Perinatal Study (discussed in Sect. 3) noted discrepancies in how the “Color” sub-score was evaluated. The authors suspected that African American babies (or, whichever babies were labeled “Negro,” excluded from the “White” category, and were not considered “Puerto Rican”) were disproportionately given scores of 9 or 10 rather than 7 or 8, a difference they supposed could be blamed on “difficulty in assessing skin color” (Jackson, 2023, p. 226; Drage et al., 1964, p. 228).^23^ While this could reflect a bias inherent to the Apgar Scoring itself, assigning artificially higher scores to more melanated babies or vice versa, it could be a reflection of a broader bias. For example, today’s widespread (albeit playfully diagnosed) notion of “wimpy white boy syndrome” (Oelberg, 2014). While seeming to target less-melanated babies with a stereotype of weakness and fragility, this “syndrome” effectively stereotypes *non-white, non-male babies* as inherently more robust and therefore not as worthy of careful attention and concern. An apparent racialized difference in scoring could be caused by an underlying bias of the medical gaze. Controlling for these external variables in a study (to eliminate racially influenced observation) is no solution, as will be discussed below within a discussion on the shortcomings of psychometric theory for evaluating patient outcome measures.

### Problems when validating patient outcome measures^24^

The role and use of the Apgar Score shifted over time, and likewise the processes of validating the Score also shifted. Today, what would it look like to re-validate the Score in terms of how well it operates for different racial groups? As discussed above, we cannot simply compare the Score distribution of, for example, African American babies with White babies and see if they differ. We already know that there are racial health inequities in the US healthcare system, so it would not make sense to make the assumption that the only difference between the two groups of babies is neonatal melanin levels. If we tried to control for systemic differences in health, comparing only newborns with similar health statuses across racial groups, we end up (by virtue of the previous subsection) back where we started: What measure of newborn health do we have confidence in besides the Apgar Score?

Even when controlling for factors like education and socioeconomic status (Kennedy-Moulton et al., 2022; Petersen et al., 2019), we know from studies of maternal mortality that other factors that are not easily measured or controlled-for can have huge health impacts on the health of mothers. Examples of these would be chronic stress (Geronimus et al., 2005), discrimination in care (Vedam et al., 2019), generational wealth, or availability of medically trained intermediaries or advocates.^25^ If the maternal environment is already at a disadvantage, babies may already be set on the wrong foot. Even studies that look at how well the Apgar Score predicts outcomes within racial groups assume that there will be no differences in these groups unrelated to physiology (Gillette et al., 2022). For example, there could be disparities in treatment by health care providers even within the same facility, or there could be a difference in maternal agency and power to advocate for oneself within the healthcare system. There could also be a difference in trust, which could impact what mothers feel they can ask for without potentially inviting negative recourse (Mohamoud et al., 2023).

This line of inquiry highlights the inadequacy of validating a clinical measure within the framework of psychometric theory, which traditionally takes for granted that the research which validates the measure can be accomplished outside of the environment in which it will be implemented. While validating measures across different demographic groups is a familiar problem in psychometrics more broadly, strategies of validation often consist of studying knowingly unrealistic samples and situations in order to control confounding variables. With some psychometric instruments, we can get by with this assumption of “in vitro” style testing. For example, a test for reading comprehension could be designed in a lab-like environment where each student taking the test has been served an adequate breakfast, had an adequate sleep duration, and the room is perfectly quiet. We know that such an environment is not likely to be repeated in implementation, but we generally consider those local variables the responsibility of those participating in the testing and proctoring to control. The fact that some students may score artificially lower than they otherwise would have due to not having had an adequate breakfast would not be considered a mark against the test’s validity.^26^ This is not an assumption we can generally take with clinical measures. It is hard to imagine what it would mean to study the performance of the Apgar Score in a perfectly controlled lab-like environment, but even if it were possible, such a study would not tell us about the performance of the Score within the actual medical system that patients experience. We cannot simply brush aside contingent variables introduced during implementation like we might in the case of a reading comprehension test, especially if the impact of contingent variables is precisely the thing we are investigating. If a measure does not tell us what we intend it to within the temporal, physical, and personnel constraints of the clinical context, it’s not a valid measure. Or, put another way, it’s not a valid *clinical* measure.^27^

This is a serious claim with interesting repercussions when combining historical with philosophical perspectives on measurement; after all, the “clinical context” of a measure itself changes over time, even shaping itself around the practice of measuring. This means that it’s rather difficult to talk about validation as a process which ever “ends.” And, certainly this implies “validation” can be in the eye of the beholder; with multiple audiences with different demands from measured data,^28^ what is required for validation can shift and regroup over time.^29^ In a sense, clinical outcome measures do not measure *outcomes*, per se; they act as measures of other things (such as interventions, techniques, whole health systems) toward achieving certain desirable patient outcomes. They cannot be validated without an understanding of the judgments or actions which will take place to achieve these outcomes, as well as the scope of outcomes meant to be analyzed (individual outcomes or group-level outcomes). Thus, the last step of validation must always be performed in implementation, by users of measures—first, by choosing to use a measure at all, and second, in justifying its use in the context of how that data will be used to inspire clinical action.

## Conclusion: measures for achieving outcomes

This case shows the complexity of coordination and validation of outcome measures. The actual coordination that we arrive at in the end is not necessarily one solely between empirical data and numerical notation. And, the target phenomena or construct that is being “epistemically iterated” can shift over time.^30^ For the Apgar Score, it was not the case that the higher-order phenomena (such as umbilical cord blood pH levels) became better and better coordinated with the Score, alongside better and better theories of how asphyxiation worked. We did in fact improve our understanding of the biochemistry of asphyxiation over the course of the latter half of the twentieth century, but instead of adjusting the Apgar Score accordingly to more closely reflect oxygen deprivation, the opposite happened. The Apgar Score was recommended to be disassociated from asphyxiation diagnoses of individual infants (AAP, 1986). What instructed us to keep the Apgar Score and discard its equivocation with asphyxiation was that it turned out that Score is a better predictor of mortality than biochemical data (like blood gas levels) are (Casey et al., 2001). The Score's clinical use and research uses, while shifting over time, are ultimately about saving infant lives and selecting health systems interventions that lead to the same. Yet, these same mortality statistics differ between racial populations due to systemic health inequities. This renders us, at present, unable to assess the Score’s validity across racial populations either from the perspective of coordination or from the perspective of psychometric validation. This case demonstrates the problems that racial inequities pose not only for the people whose lives are affected by prejudice, but also for the two dominant theories in philosophy of measurement for how meaningful measures are achieved in science.

Are philosophers capable of assessing or guiding clinical outcome measures in the future? Questioning the value of the representational view of measurement for clinical measures is a good start. Seeing the activity of measurement as aiming to arrive at coordination of notation with the unique structure of an empirical property encourages the erroneous hope that a validated outcome measure will be equally good for any purpose. This kind of assumption is what enabled the ecological fallacy of explaining individual children’s behaviors and neurological conditions by associations derived from group-level analyses of Apgar Scores. For individuals who are already marginalized in a health system, this can be an especially dangerous error. While the harm from white-centric language of the “Appearance” item of the Apgar Score may be comparatively easy to ameliorate in the future, the past and present danger of racialized children being targeted by pseudo-medical explanations related to data collected at their birth deserves further historical attention and sociological investigation. Adopting an extended view of the “problem of coordination” (Barwich & Chang, 2015) which includes coordination with the clinical judgments and actions that take place because of measures could be a helpful way forward (Jackson, 2023, p. 272–275). An extended view of the coordination process could allow us to see patient outcome measures as *measures for achieving outcomes*, which will help us guard against fallacious reasoning that results from believing that coordination can or should occur prior to measurement use. This resembles another proposal for amending the term “validity” to “validity of action” (Truijens et al., 2019). Leah McClimans’ processual account for working towards construct validity in patient outcome measures, “epistemic dialogue,” could easily be compatible with this extended view, although I believe it is intended apply to the original problem of coordination (McClimans, 2024).

One possible way to prevent some “mistranslational” abuses is return to something like what Virginia Apgar originally intended: the Score as a systematic way of coding clinical observation into loose categories (poor, fair, good) that are meaningful enough for quick clinical assessments and broad research claims. A numerical Apgar Score should never follow a child later in life. In longitudinal studies, these broader categories can still be used to categorize outcomes. We should preserve the best aspects of “small data,” the kind of measuring that is relevant to the people in the room, while being wary of creating opportunity for future mistranslations and operational slippages when this data is transformed into “big data.” A similar argument has been made for why the measurement of cervical dilation in *centimeters* should be abolished and replaced with a scale which is ordinal, with fewer, vaguer, values (Jackson, 2025). This is a kind of coordination which is guided by ethics (Rodriguez Duque et al., 2024), not just epistemology, of measurement.

In addition to this case showing how better measurement is about better coordination with a landscape of decision-making, not always about more precision in the traditional sense, this kind of normative work opens up a way that philosophers of measurement can contribute to conceptual engineering of measuring scales employed in medicine.^31^ As health data systems become more integrated,^32^ and data which is gathered for one purpose finds their way into unexpected places, it becomes more pressing to understand validity not as an inherent property of an instrument but *as an argument* about how and when we can use a measure (Kane, 1992). Philosophers should not shy away from the challenge of providing relevant guiding theories for whether and how the information we get from clinical indices (like the Apgar Score) should be preserved for future use or kept within the bounds of a particular context, either by reengineering data format and scale, storage practices, or access (data governance). Data which are produced by processes taken to be inherently valid, and thus made equally available for all clinical purposes, are ripe for misuse within algorithmic systems which are increasingly permeating epidemiological and clinical spaces (BMA, 2024).

In order to do this work, historical methods, and philosophically-informed historians, have an essential role to play. To guide a measuring practice, we must first understand it. In the case of Apgar’s scoring method (and the eventual “Apgar Score” it became), we must first understand that truly there are two scores: the one-minute score (for clinical attention) and the five-minute score (which also serves research purposes). Understanding the physical environments in which a measuring practice was shaped, the social and institutional situations and demands it has adapted to fulfill,^33^ the details of measuring practices over long periods of time, showing how scales first arise and how their changing uses over time shift the way data is prefigured and interpreted, is essential for tracing how validation (and re-validation) can and should occur.

## Appendix

See Table 2.

**Table 2:** Apgar Score obstetric uses, scales of application and analysis over time

Apgar Score: obstetric uses over time					
	Origins: **1949–52** “Scoring method,” assessed at 1-min	Late-1950s–mid 60 s Assessed at 1-min and 5-min	Collaborative perinatal study (1959–65)* Assessed at 1-min and 5-min	Mid-1960s–90 s Assessed at 1-min and 5-min	Best practice today Assessed at 1-min and 5-min
Use(s)	Evaluating impact of anesthetic techniques Testing whether oxygen deprivation levels were normal (randomly distributed in population) or pathological	Mediator of risk assessment (for groups of newborns) Score thresholds used as rule-of-thumb for clinical guidance (prompting observation of individual newborns)	Assessing association between newborn asphyxiation after birth with risk of later childhood morbidities	Assessing causal association between asphyxiation and later mental and physical morbidities (for groups of newborns) **Score thresholds used to determine clinical actions (for individual newborns)** **Explaining unfavorable social behaviors (of individuals)** **Predicting likelihood of future health outcomes (of individuals)**	5-min Score used to assess the success of interventions and care systems 1-min Score used to approximate level of care provider concern and to direct clinical attention and resources in the moments after birth
Scale of application and/or analysis	Groups of newborns	Groups of newborns (in risk assessment) Individual newborns (used to guide clinical attention)	Groups of newborns	Groups of newborns (in research on morbidities) **Individual newborns (to direct clinical decisions after birth)** **Individual newborns (in prognostics and diagnostics)**	5-min Score: Groups of newborns (in epidemiological research) 1-min Score: Individual newborns (where used as a clinical guide)

*1-min Score found to have poor association with morbidities compared to 5-min Score in this study; it was nearly dropped as a result (discussed in Sect. 2)

The bolded text indicates uses and applications which fully commit the ecological fallacy (or “G2i inference” failure), as discussed in Sect. 2

## Footnotes

1 As far as I am aware, this term was coined by Marion Boulicault (2022). The area of literature to which it refers examines cases of measurement and critiques the instruments, actors, theories, and inherent assumptions through the lens of how these practices impact marginalized groups (e.g., using feminist theory to examine the sexist assumptions underlying fertility measuring practices (Boulicault, 2023)). Such studies which focus on the role of physical measuring tools in ossifying marginalization (e.g., Braun, 2014) can be considered as a part of (and inspiring) a broader interest in the role of seemingly innocuous material objects in reinforcing and anchoring systems of oppression (Liao & Carbonell, 2023; Liao & Huebner, 2021). Other works that could be considered to influence this growing area of scholarship would be classics such as Gould’s work on IQ testing and craniometry and Porter’s work on the rhetorical role of quantification in conveying objectivity and removing a sense of responsibility (Gould, 1981; Porter, 1995). This present paper represents the possibility of going further with the use of historical and sociological cases of measurement to question the conceptual foundations of philosophy of measurement itself, i.e., letting history of measurement play a role in critiquing philosophy rather than only the other way around. When applied to work that is critical of traditional concepts that normally go unquestioned in philosophy of measurement (e.g., precision, standardization, accuracy, validity, specificity), I prefer the term “critical measurement studies” to reduce confusion with the term “metrology” being associated with the study of physical weights and measures. I thank an anonymous reviewer for requesting that I situate the present paper within this growing and important discourse.

2 In this article, I focus only on obstetric uses of the Apgar Score. In pediatric guidance, the Apgar Score is also used for cooling decisions, wherein the 10-min Score is relevant (Brigham & Women’s Hospital, 2021; Northern Neonatal Network, 2021). In addition to considering other qualifying factors, pediatricians may determine whether therapeutic hypothermia is an appropriate course of treatment by whether the infant scores below a “5” at 10 min. Future work is necessary for understanding the Apgar Score in the pediatric context (and specifically, how it influences clinical decision-making within the neonatal intensive care unit), to better understand the full scope of clinical decisions which may hinge sensitively on a single Score point difference.

3 Note that this guidance pertains to the “term infant and late-preterm infant” (ACOG, 2015; ACOG & AAP, 2014).

4 While “psychometrics” may sound to only pertain to psychological phenomena, it is generally considered a broader discipline which applies statistical tools and techniques to measure phenomena in educational, sociological, and clinical applications as well as psychological traits and states. Apgar’s score was not crafted with psychometric techniques historically, but today, if such a score was proposed it would certainly be expected to show its validity and reliability in psychometric terms in order to gain legitimacy in scientific and medical circles.

5 Psychometric validation consists of a whole host of subtypes and strategies, including content validity (interpretability and relevance of individual items), criterion validity (agreement with other trusted measures), construct validity (reflection of trusted theoretical knowledge), and face validity (agrees with common-sense understandings) among others. Over the course of the twentieth and twenty-first centuries, different theoretical paradigms for what psychometric validity pertains to have been introduced (Edwards et al., 2018) (i.e., whether it is the property of a test, a property of an inference, or a property of an argument). Elements of each of these traditions linger in current practices.

6 Neonates were not the only vulnerable thing on Apgar’s mind during this time; Anesthesiology itself was still a nascent and under-funded area of medical research, struggling for air. She saw an opportunity to carve out neonatal resuscitation as an area of expertise and responsibility that anesthesiologists could take on, growing the territory her specialty could claim.

7 Portions of this paragraph are borrowed or summarized from Jackson, 2023, p. 185.

8 It is surprisingly difficult to find quantitative evidence of how often babies who will eventually develop deep skin tones begin life outside the womb with high melanin levels. One observational study which used a bespoke 1–4 scale for visually classifying neonatal skin tones (with 1 being described as white/pink, 2 as pink/beige, 3 as light brown, and 4 as brown) found that the distribution of 63 African American newborns across the four categories was n = 5, n = 19, n = 20, and n = 19 respectively (see Fig. 1 for skin tone scale reference images, p. 608) (Maya-Enero et al., 2021). But, they note that they expanded the study period (originally 1 year) by 17 months to recruit additional newborns that would fit the 4 (“brown”) category. It is thus difficult to say even broadly what proportion of newborns of a given racial group (such as African American) may appear to have higher initial melanin levels.

9 Mental test scores (earned or imagined), as well as physical measurements, served as evidence of intellectual disability and therefore propensity toward criminality (Allen, 1986, p. 240). Thought to be a hereditary trait, assessments of mental deficiency could also extend to the children (or potential future children) of those deemed feeble-minded.

10 For an excellent overview of different twentieth century biological and social theories for how maternal behavior impacted the capacities of their children, some racist and only thinly disguised as scientific (e.g. the myth of “crack babies” in the US), see Richardson (2021). See also Roberts (2002), especially p. 61–67, for the social context of why Black women in the US have been repeatedly stereotyped as “unfit” mothers, a politics of controlling parental rights which has its earliest roots in enslavement.

11 This example comes from a personal experience shared with me by an American colleague after a talk I gave on the history of the Apgar Score; I owe her thanks for pointing me towards a more critical examination of how the Apgar Score has impacted individuals later in life, long after its clinical role had been served.

12 It should be firmly stated that “race” and skin tone variations do not equate. For an overview of the complexity of philosophical positions on race, see Glasgow et al. (2019). My core argument in this paper does not ultimately depend on whether or not we take “race” to be biological, cultural, political, social, or another kind of category. Historically, in the United States, there has been much overlap between colorism and racism. That said, any population can be racialized by others, and this racialization can be linked to a variety of attributes that may or may not be directly visible.

13 The term “asphyxia” broadly refers to both causes and effects of oxygen deprivation, namely “hypercarbia (increase in carbon dioxide, leading to acidosis) and hypoxemia (low oxygen level), and the eventual metabolic acidemia (build-up of acid which negatively impacts the heart) that results from these two conditions” (Jackson, 2023, p. 237).

14 Elina Vessonen has articulated the “psychometric theory of measurement” as having a status as a competing (but in her view, actually complementary) philosophical framework to the representational theory of measurement (Vessonen, 2017). For my purposes, whether these frameworks are truly competing or complementary has no impact.

15 The beginning of RTM as a formal theory is often credited to the work of Suppes (Scott and Suppes, 1958; Suppes, 1951) leading up to the three volumes of *Foundations of Measurement* (Krantz et al., 1971; Luce et al., 1990; Suppes et al., 1989). For a historical overview of RTM, see Savage and Ehrlich (1992).

16 Less charitably interpreted, RTM’s set-theoretic model of measurement, which connects sets of “prefigured” elements, gives the illusion of simplicity where it is most necessary for philosophers to understand the process of individuation and representation as “purposive,” not mind-independent, and “carried out to facilitate the achievement of certain aims” (Chang, 2022, Ch. 2: “Correspondence”).

17 There are many technical details of RTM that could be mentioned here but are not necessary for my argument. RTM’s achievement of a homomorphism (the successful coordination) is typically associated with meeting two requirements: existence (of a many-to-one mapping from the empirical set to a representational set) and uniqueness (of that mapping—meaning there is not another richer representational set we could have chosen). The strength of these requirements is their abstraction; they are meant to apply to any measuring domain and are agnostic to purpose. Their weakness is the same; these requirements do not aid us with understanding when the coordination process has been achieved for domain-specific measures like clinical outcome measures.

18 It is worth pondering whether we have not actually seen many bifurcations of the temperature concept for different purposes; see Jackson (2023) for examples of problem contexts in which non-interval scale instruments for temperature are favored (p. 269–270).

19 What I mean by “outside of the measuring process” is outside of the idealized model of the measuring process—the measuring process before it encounters individual subjects or instances. One rather extreme example of the role of auxiliary theories in patient-centered measurement in medicine is the use of the “open drop method” for anesthesia in the early twentieth century (Jackson, 2021). This coordination, between the correct amount of ether for the individual patient with the correct drop rate throughout the procedure, was accomplished without numerals by paying attention to the individual patient’s physiological signals.

20 The measurement of sapphire quality is an example of physical measurement which is aided by a strong auxiliary theory (Collins, 2001). The procedure for measuring “Q” (quality) was extremely sensitive and difficult, and yet whenever a higher number was achieved, this datapoint was known to be more trustworthy than whatever lower estimates had been obtained for the same sapphire. This is because any flaws in the procedure (such as the sting holding up the sapphire absorbing some of the energy) would have caused a falsely *lower* estimate of energy dissipation, not higher.

21 What I mean by “higher-order” is mostly an intuitive understanding of evidentiary directionality—“higher” meaning it acts at a meta-level relative to another measure. For example, the number of infant deaths that occur for infants who receive a certain Apgar Score could be considered supporting or non-supporting evidence for the Score’s approximation of overall infant well-being or mortality risk. Contrastingly, we would not expect for the Apgar Score of an infant to serve as evidentiary support of whether the infant is currently dead or alive, or the Apgar Score of a group of infants to support or challenge the number categorized as “dead.” Thus, deaths here would be acting as “higher-order” evidence relative to Apgar Score.

22 Face validity is a psychometric term for the extent to which a measure appears, on its face, to measure what it intends to measure. Some specific uses will affect whose subjective perception is relevant for assessing face validity; here I mean to convey a kind of common-sense trust from those using and interpreting the measure.

23 Several sentences in this paragraph first appeared in identical or similar forms in Jackson, 2023.

24 Several paragraphs in this subsection appeared in identical or substantially similar forms within Sect. 7.3 (pp. 245–248) of Jackson, 2023.

25 The likelihood of personally knowing or being related to a medically trained professional who can serve as consultant and advocate is a hidden class privilege. For more on the importance of medical advocacy, especially for African American women navigating the US medical system, see Molina et al. (2015).

26 This is not to suggest that that educational testing specialists and policymakers do not seriously consider the relationship between testing conditions and testing outcomes. Yet, the likely intervention would be to ensure that all students have access to adequate breakfast; understandably, the response would not be to attempt to change the test to be insensitive to whether a student has had breakfast.

27 We may as well design an instrument for measuring the depth of the ocean that only works within the moon’s gravitational field. A measuring practice that cannot be practiced in the environment in which it is intended to be used is a poor one.

28 For a discussion of the role of “epistemic audiences” in measurement, see Jackson (2021).

29 For example, Truijens et al. (2019) has also pointed out the distance between psychometric studies and clinical applications, even clinical research applications, arguing that “the epistemic goal for applied researchers is principally different from the psychometric goal of the test developer” and that “*psychometric validity* is insufficient in covering the question of what constitutes a valid epistemic process in psychotherapy research” (p. 8 and 7, emphasis theirs). They point out the flaws of using a diagnostic tool (e.g., the Beck Depression Inventory, or “BDI”) as a patient outcome measure without acknowledging or justifying this decision. This involves using BDI (which is intended as an operationalization of “depression symptoms”) to function as an operationalization of “depression severity over time,” which itself then functions as an operationalization of “treatment efficacy” (p. 3 and 7). In the course of these unjustified leaps, BDI’s target for coordination transforms: from an individual patient’s current symptoms, to a group of patients’ symptom *changes* over time, to an evaluation of a treatment as effective or not effective.

30 This shows that we can have “moving targets” (Hacking, 2007) even when scoring neonates who have never been measured before. The movement here comes not from people’s own changing perceptions of themselves or others as embodying a category, but from the shifting understandings of the way that clinical data matters for informing judgments and comparisons.

31 By “conceptual engineering” in this context, I mean normative philosophical work which hopes to “change the conscious or unconscious inferences that we draw about the [data] we encounter” (insertion mine; Isaac et al., 2022, p. 5).

32 For example, in the UK, the National Health Service (NHS) is in the process of moving towards a “single patient record,” which means that all health data (including infant data) would be accessible to all care providers (and other relevant government entities) throughout the life-course (NHS 2025).

33 For a discussion of the role of contexts-of-use in measurement, or “ecological niches,” see Jackson (2023), particularly pp. 248-52 and 268-75.
